# Single Dose Caffeine Protects the Neonatal Mouse Brain against Hypoxia Ischemia

**DOI:** 10.1371/journal.pone.0170545

**Published:** 2017-01-27

**Authors:** Max Winerdal, Vijay Urmaliya, Malin E. Winerdal, Bertil B. Fredholm, Ola Winqvist, Ulrika Ådén

**Affiliations:** 1 Department of Women´s and Children´s Health, Karolinska Institutet, Stockholm, Sweden; 2 Department of Physiology and Pharmacology, Karolinska Institutet, Stockholm, Sweden; 3 Department of Medicine, Karolinska Institutet, Stockholm, Sweden; Universidad de Castilla-La Mancha, SPAIN

## Abstract

In this randomized blinded study, we investigated caffeine 5 mg/kg treatment given directly after neonatal brain hypoxia ischemia. Brain morphology, behavior and key brain infiltrating immune populations were examined. Caffeine treatment significantly improves outcome when compared to phosphate buffered saline. Flow cytometric analysis of immune responses revealed no persistent immunological alterations. Given its safety caffeine emerges as a candidate for neuroprotective intervention after neonatal brain injury.

## Introduction

Neuroprotective strategies are needed for neonatal hypoxia ischemia brain injury (HI). Mild hypothermia is currently the only available treatment in widespread clinical use [1], though difficult to achieve in low income countries. Thus, an affordable, technically uncomplicated alternative is highly desirable. When caffeine was given to treat apneas cerebral palsy significantly diminished [2], suggesting that caffeine might be a candidate for the treatment of brain HI.

Much is published concerning the risk of adverse effects of caffeine use. Dosage is of major importance since caffeine is a competitive inhibitor of adenosine A_1_, A_2A_ and A_2B_ receptors at lower doses whereas higher concentrations inhibit phosphodiesterase, responsible for many of the adverse effects reported [3]. Available prospective randomized studies of perinatal use have been unable to show adverse effects of clinically relevant doses of caffeine [3].

We investigated morphological, behavioral and immunological outcomes after HI in neonatal mice randomized to caffeine or phosphate buffered saline (PBS) treatment. Since the nature of caffeine's immunomodulatory effects has been questioned [4], a broad spectrum of brain and spleen immune cell populations were investigated.

## Material and Methods

All experiments were approved by the regional ethics committee, Stockholms norra djurförsöksetiska nämnd, in accordance with local institutional guidelines and the Directive 2010/63/EU.

### Hypoxic ischemic brain injury

The modified Vannucci model was used and evaluated with morphology and behavioral tests. There was no mortality, severe illness or need for early euthanasia. WT C57/bl6 specific pathogen free (SPF) mice (Charles River Laboratories) of either sex were used, with free access to pelleted food and housed in open cages with standard enrichment with daily monitoring of the animals in accordance with local institutional guidelines. Pups were kept with their mother at all times except during surgery and hypoxia. Randomly allocated (https://www.random.org), single-dose caffeine 5 mg/kg or PBS was administered *i*.*p*. directly after HI (n = 29 and n = 13 respectively). Evaluations were performed by investigators blinded to treatment. Animals were sacrificed by injection of 240 mg/kg sodium pentobarbiturate *i*.*p*. and cardiac perfusion with 12 mL phosphate buffered saline (PBS) to remove intravascular blood cells.

HI was performed at postnatal day 10. Unilateral electrocoagulation at 8 Watt of the right carotid artery was carried out via midline neck incision under isoflurane sedation and local bupivacaine infiltration to minimize pain and distress. Pups rested one hour with the dam after surgery, prior to one hour of 10% O_2_ in 90% N_2_ at 36˚C skin temperature. During sham operation the carotid artery was visualized as described above but not electrocoagulated.

### Immunohistochemistry

Brains were collected after behavioral evaluation in the Caffeine or PBS treatment groups (n = 29 and n = 13 respectively) at postnatal day 24, two weeks after HI. In accordance with previous studies [5], three levels of each brain were collected, corresponding to bregma 1.3 mm, -1.64 mm and -2.92 mm in the adult mouse brain using cryostat 10–12 μm sections. Tissue sections were fixed for 10 min in 4% paraformaldehyde. Endogenous peroxidase was blocked by 0.3% H_2_O_2_ in 3% Normal Horse Serum for 10 min. Microtubule associated protein-2 (MAP-2) staining VECTOR® M.O.M.™ Immunodetection Kit and VECTASTAIN® Elite ABC-Peroxidase Kit were used according to the manufacturer’s specifications. The enzymatic coloration of immunoreactivity was performed by simultaneous full immersion in 3,3´-diaminobenzidine, DAB for all slides. Imaging was performed with a Nikon eclipse E800 microscope with an Olympus DP70 camera with DP controller, and version 3.1.1.267 acquisition software. Percent MAP-2 stained area and infarction size were manually delineated using Adobe Photoshop version 12.0.4 in brain slices with investigators blinded to genotype and treatment. Atrophy was calculated as the remaining tissue loss not described by the infarction area delineated by loss of MAP-2 compared to the uninjured hemisphere using the formula 1-(Damaged hemisphere area including infarction area)/(Undamaged hemisphere area) [6].

### Behavioral tests

Behavioral assessments were done at 24 days age, two weeks after HI in caffeine or PBS treated animals (n = 29 and n = 13 respectively). Open Field test (Kungsbacka Mät- och Reglerteknik AB, Fjärås, Sweden) was used to investigate explorative behavior during 30 min. The animals were taken directly from their home litters and put in the open field box without further handling. In addition, the beam walking test, where mice walk three times back and forth on a beam (10 mm wide, 600 mm long), was performed directly after the open field test. Mice were handled with care to assure a calm explorative state of mind in the animal, and the number of slips with the hind limbs recorded by investigator blinded to treatment group.

### 2.6 FACS analysis

Randomization to caffeine or PBS treatment and sham or HI operation was performed at postnatal day 10 and samples were analyzed at 24h, 72h and 2 weeks after HI (total n = 52, treatment allocation is discernible in Fig 2).

Damaged and undamaged brain hemispheres were gently homogenized using a loose fit glass homogenizer, and cell suspension passed through a 100um cell strainer prior to staining with fluorochrome conjugated antibodies. Splenocyte cell suspensions were prepared by gentle homogenization through a 100um cell strainer followed by red blood cell lysis with ACK buffer for 5 min at room temperature. For antibodies used see S2 Table. Flow cytometric data was collected on a FACS Aria (BD Biosciences). 2.5x10^6^ events were collected from each brain hemisphere.

A data driven gating strategy was used with the FlowCore package for R [7] as previously described in detail [8]. Debris was manually removed using a polygon gate in forward and side scatter. A curvfilter was used to filter out immune cell populations. Dead cells were excluded using DAPI stain. Negative population was selected as reference and cells with higher expression than that were regarded as positive cells. Median fluorescent intensities of the selected populations were used to discriminate between positive and negative populations. The same filter settings were used for each marker in all brain samples. After automated batching, all gate settings were visually confirmed and no manual adjustments were required.

### Statistical analysis

Mann-Whitney U test was used to analyze data on morphology and behavior data. Statistical calculations were made in StatSoft, Inc. (2011) STATISTICA, version 10. Heatmaps were made in R version 2.15.

## Results

**Fig 1:**
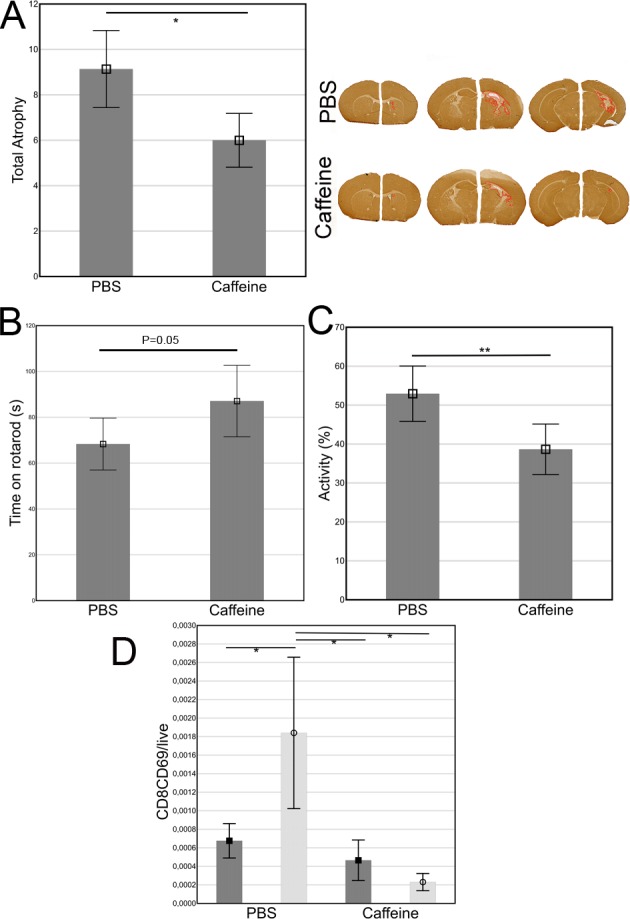
Improved outcome in caffeine treated animals compared to controls (n = 29 and n = 13 respectively). A) Brain infarction was delineated by MAP-2 and atrophy calculated as 1-(Damaged hemisphere area including infarction area) / (Undamaged hemisphere area). The depicted brains are from the animals with injury size close to mean in each group respectively. B) Time spent on the Rotarod. C) Activity and velocity in the open field test was significantly decreased in the caffeine treated group. D) The only significantly altered population after caffeine compared to PBS treatment. Dark bars and squares represent sham operation and light bars with circles HI.

A single dose caffeine 5 mg/kg administered directly after neonatal HI significantly decreased atrophy by 44% (p<0.05) defined by MAP-2 staining compared to PBS treatment (Fig 1A), paralleled by a borderline significant (p = 0.05) increase in time spent on the Rotarod (Fig 1B), indicating better balance and coordination. In addition, open field test activity significantly decreases after caffeine treatment compared to controls (p<0.01), signifying improved habituation (Fig 1C). Thus, caffeine given directly after HI is neuroprotective since improved neurological outcome was demonstrated in treated neonatal mice.

**Fig 2:**
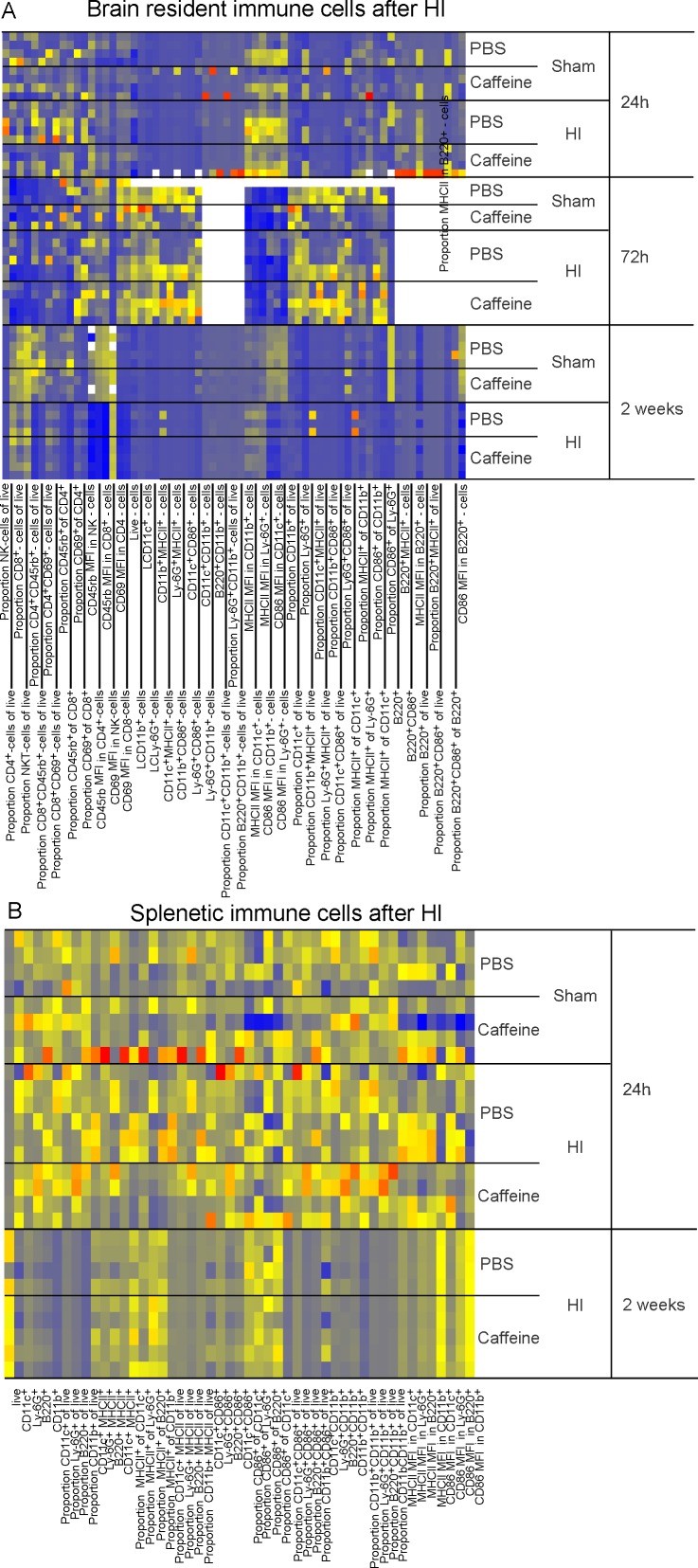
Heat map of all investigated immune populations after sham operation or HI 24h, 72h and two weeks after randomization to caffeine or PBS (n = 3–5 in each group, total n = 52). The data was normalized to zero for the lowest value and one for the highest value in each variable with zeros in blue through yellow to ones values in red. Whites are missing values. MFI = Median florescence intensity.

Local and systemic immune populations investigated 24h, 72h and two weeks after HI, revealed surprisingly few alterations considering the decrease in atrophy and improved outcome (Fig 2). Only the activated CD69^+^CD8^+^ T-lymphocyte population was significantly reduced in the brain 24h after caffeine treatment compared to PBS (Fig 1D). No differences in immune population frequencies or activation status were found in the brain 72h or two weeks after HI.

Systemic effects were investigated simultaneously in spleen. No significant differences were seen between PBS and caffeine treatment animals at any time.

## Discussion

Caffeine protects the neonatal mouse brain after HI, as observed by decreased brain atrophy and behavioral correlates in altered motor function and activation in the open field test. This confirms the neuroprotective properties of caffeine reported in humans [2] [9]. Of note, caffeine could potentially be administered trough breast feeding since prenatal continuous maternal caffeine intake reduces brain damage after neonatal HI in rats [10].

Of all immune populations investigated, only activated brain CD8^+^CD69^+^ T-lymphocytes 24h after HI differed significantly between caffeine and PBS. This indicates an immunomodulatory effect while caffeine is present with no apparent long term consequences. Since caffeine breakdown is slower in infants than adults, a sufficient caffeine concentration could be expected for as long as 24h but hardly for 72h or two weeks [3]. Admittedly, immune effects able to influence outcome are possible earlier than 24h after brain HI. However, our primary concern in this study was long term alterations, since the fear of persistent adverse effects have hampered the proceeding of caffeine as a neuroprotective agent.

With reference to mild or no persistent adverse effects, simplicity of administration and favorable pharmacokinetics we argue that studies addressing the therapeutic use of caffeine for neuroprotection after neonatal HI in larger animals prior to controlled prospective randomized clinical trials are urgently warranted.

## Supporting Information

S1 Fig Magnification of MAP-2 staining of brain. (TIF)

S1 Table Flow cytometry dataset. (CSV)

S2 Table Antibodies used. (DOCX)
