# Supplementary figures and images for: Single Dose Caffeine Protects the Neonatal Mouse Brain against Hypoxia Ischemia
Source: PLoS One. 2017 Jan 27;12(1):e0170545. doi: 10.1371/journal.pone.0170545 (PMC5271335; doi:10.1371/journal.pone.0170545)

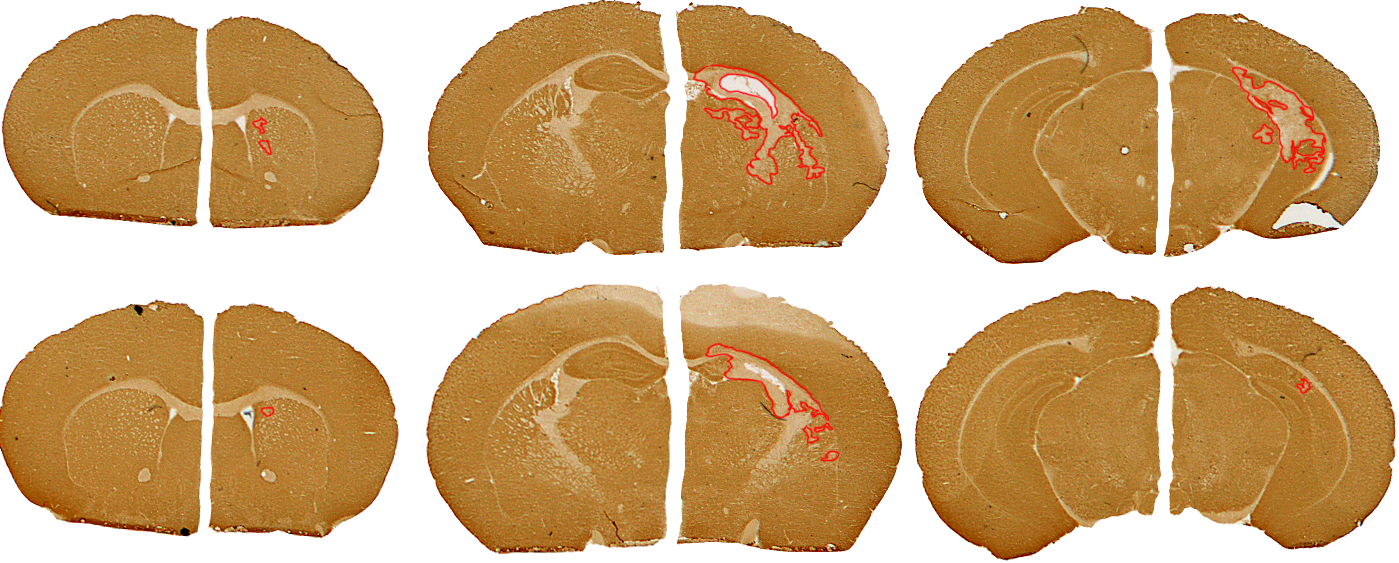

Supplement: S1 Fig — (TIF) [file pone.0170545.s001.tif]
