# Supplementary material for: Single Dose Caffeine Protects the Neonatal Mouse Brain against Hypoxia Ischemia
Source: PLoS One. 2017 Jan 27;12(1):e0170545. doi: 10.1371/journal.pone.0170545 (PMC5271335; doi:10.1371/journal.pone.0170545)
Supplement: S2 Table — (DOCX) [file pone.0170545.s003.docx]

| **Lymphocyte panel** | | **Product no** | **Clone** | **Species** | **Company** |
| --- | --- | --- | --- | --- | --- |
| CD8 | FITC | 100706 | 53-6.7 | Rat IgG2a | Biolegend |
| CD45rb | PE | 103308 | C363-16A | Rat IgG2a, k | Biolegend |
| NK1.1 | PerCp-Cy5.5 | 108728 | PK136 | Mouse IgG2a | Biolegend |
| CD69 | PE-Cy7 | 552879 | H1.2F3 | Armenian Hamster IgG1 | BD Bioscience |
| CD4 | APC | 100516 | RM4-5 | Rat IgG2a | Biolegend |
| TCRb | APC-Cy-7 | 109220 | H57-597 | Armenian Hamster IgG | Biolegend |
|  | DAPI |  |  |  |  |
| **Innate immunity panel** | | | |  |  |
| Ly-6G | Alexa Fluor 488 | 127606 | 1A8 | Rat IgG2a | Biolegend |
| H2iab | PE | 116408 | AF6-120.1 | Mouse (BALB/c) IgG2a | Biolegend |
| B220 | PE-TR | 551489 | RA-6B2 | Rat IgG2a, k | BD |
| CD11b | PerCp-Cy5.5 | 101228 | M1/70 | Rat IgG2b, k | Biolegend |
| CD11c | PE-Cy7 | 117318 | N418 | Armenian Hamster IgG | Biolegend |
| CD86 | APC | 105012 | GL1 | Rat IgG2a, k | Biolegend |
|  | DAPI |  |  |  |  |

**Supplemental table 1** List of antibodies.
